# A Human-in-the-Loop Reference-Label Curation Framework for Task Classification During Free-Living, Using 3D Animation of IMU Data

**DOI:** 10.3390/s26216683

**Published:** 2026-10-22

**Authors:** Ryan Riddick, Raimundo Sanchez, Paul Hodges

**Affiliations:** Centre for Innovation in Pain and Health Research, School of Health and Rehabilitation Sciences, University of Queensland, 84a Services Rd, St Lucia, Brisbane, QLD 4067, Australia; r.sanchez@uq.edu.au (R.S.); p.hodges@uq.edu.au (P.H.)

**Keywords:** inertial measurement units, task classification, machine learning, reference standard, data curation, animation, repeatability, agreement, biomechanics

## Abstract

**Highlights:**

**What are the main findings?**

- Reviewers labelling IMU-derived skeletal animations agreed substantially with their own video labels (the reference standard): the macro-F1 score was 61.9% (Cohen’s κ = 0.67) for the detailed label set and 78.0% (κ = 0.74) for a simplified, four-class taxonomy.
- The dominant source of disagreement was the classification of transition states, and this pattern held even when reviewers compared video against video—indicating that it reflects an inherent ambiguity in movement boundaries rather than a shortcoming of the animation method.

**What are the implications of the main findings?**

- IMU-derived skeletal animation review may offer a privacy-preserving alternative to continuous video for generating reference activity labels in free-living settings, since it avoids capturing identifiable imagery while still enabling human annotation that agrees substantially with video-based labels in a simulated living environment. Validation in free-living recordings is still required.
- The label taxonomy should be matched to the intended use case: broad categories (sitting, standing, stepping, lying) yield substantially higher reliability than fine-grained labels, which may better suit applications needing only coarse activity discrimination.

**Abstract:**

Reference-labelled data for training activity-recognition models from wearable inertial measurement units (IMUs) are typically collected in laboratories, which poorly represent real-world movement, and often use video, which raises privacy concerns and does not scale to long recordings. This study evaluated a novel human-in-the-loop annotation framework in which IMU-derived skeletal animations are reviewed by human raters to generate activity labels. Eight participants performed 72 functional tasks in a simulated living environment while wearing IMUs, with synchronized video serving as a reference standard. Data from five participants were analyzed. Two reviewers labelled both IMU animations and video recordings, and intra- and inter-reviewer agreement were compared across a detailed label set and a simplified four-class taxonomy. Using the video annotations as the reference standard, the intra-reviewer macro-averaged F1 for the detailed label set was 61.9 ± 13.5% (Cohen’s κ = 0.67 ± 0.12), and 78.0 ± 11.5% (κ = 0.74 ± 0.13) for the simplified taxonomy. In a sensitivity analysis excluding samples labelled as transitions, macro-F1 was 69.4 ± 10.9% and 92.8 ± 5.3%, respectively. Inter-reviewer agreement for the detailed label set was lower when labelling animations (κ = 0.51 ± 0.06) than video (κ = 0.72 ± 0.02). A main source of disagreement between reviewers was in the labelling of transitions of state, reflecting in part the ambiguous nature of defining their endpoints. These findings support skeletal-animation review as a potential privacy-preserving alternative to video annotation for generating reference labels in simulated living; validation in free-living recordings, and of the reconstructed kinematics, is still required.

## 1. Introduction

Wearable inertial measurement units (IMUs) have created new opportunities to quantify human movement continuously in natural environments. Unlike laboratory motion-capture systems, IMUs are portable, inexpensive, and capable of recording behaviour across hours to weeks in homes, workplaces, and community settings. This has driven growing interest in applying machine-learning methods to classify postures, movements, and daily-life tasks from IMU signals. These studies include general scientific inquiries into the biomechanics of locomotion [1], symptom-related behaviours like falls in populations with limited mobility [2], and use part of a preventative tool to help reduce injury in physically demanding work environments [3]. Accurate classification aids these pursuits by providing probabilistic estimates for how frequently a person adopts and transitions between types of physical tasks (walking, sitting, sleeping, etc.). These labels also help segment and contextualize the recordings into more manageable pieces, which is important because the data can span days, weeks, or even continue indefinitely.

A critical limitation to progress in this field is the scarcity of high-quality reference-labelled data for training and validating activity-recognition models in real-world environments. Most existing models are trained using laboratory datasets in which participants perform prescribed tasks under controlled conditions while labels are assigned from study protocols, direct observation, or synchronized video [4,5,6]. Although these approaches provide precise labels, they may poorly represent the variability of natural human behaviour. Movements performed in laboratories are influenced by instruction, observation, constrained environments, and limited task duration. Real-world biomechanics are shaped by fatigue [7], multitasking [8], furniture design [9], terrain [10], and social interaction [11]. Consequently, models trained on laboratory data often show reduced generalizability when deployed in free-living settings.

Several approaches have been used to generate reference labels outside the laboratory, but each has limitations. Continuous video recording is commonly considered the reference standard because it permits direct human annotation of behaviour. However, video-based methods are difficult to implement over long durations, require substantial storage and annotation effort, and raise major privacy concerns in private or occupational settings. Self-report diaries or ecological momentary assessment are scalable alternatives, but they provide coarse labels, are vulnerable to recall bias, and rarely capture precise timing of posture transitions or short-duration tasks [12]. Direct in-person observations can provide rich contextual labels, yet it is resource-intensive, intrusive, and itself may alter behaviour through the Hawthorne effect [13]. Inclusion of devices such as smart furniture [14] or pressure insoles [15] can support automated labelling, but requires specialized equipment which can increase expense and is restricted to specific settings, limiting ecological validity.

These limitations are particularly relevant for biomechanics research. Many clinically meaningful behaviours—such as repeated bending, prolonged sitting posture, guarded movement, asymmetrical loading, stair negotiation, or movement variability during pain flare-ups—occur unpredictably across diverse contexts and over prolonged time scales. Such behaviours are difficult to reproduce authentically in laboratories and impractical to capture comprehensively using video or observer-based methods. While there are efforts to use graphical models to automatically construct labels from IMU data [16], these methods still ultimately rely on data captured in controlled environments which may lead to overfitting and therefore errors when asked to predict labels in new contexts, and without the ability of a human to directly review and evaluate the validity of the labels. As a result, current annotation paradigms often fail to provide scalable, privacy-preserving, and human-reviewed reference labels for real-world IMU datasets.

To address this problem, we developed a novel human-in-the-loop annotation framework based on skeletal animations reconstructed directly from worn IMUs. Sensor fusion estimates body-segment orientation, which is rendered as a time-synchronized animated skeletal avatar for reviewer annotation. This approach preserves the movement information necessary for task classification while avoiding capture of identifiable video imagery. Because labels are generated from the same sensing modality used in deployment, the method may also reduce mismatch between training data and real-world application.

The purpose of this study was to evaluate whether annotation of IMU-derived skeletal animations can provide labels that agree with video-based reference labels for human activity recognition. Using a simulated living environment with synchronized video and IMU recordings, we compared labels derived from skeletal animations against video-based annotation, quantified inter-reviewer agreement, and examined the effect of label specificity on classification reliability. We hypothesized that this approach could, after further validation, provide a privacy-preserving alternative to conventional methods for generating reference labels in free-living biomechanics research. We then explored how the type and specificity of tasks, transitions between tasks, and the prescription of a definitional task hierarchy can affect agreement and repeatability.

## 2. Materials and Methods

### 2.1. Experiment—Simulated Living

**Figure 1:**
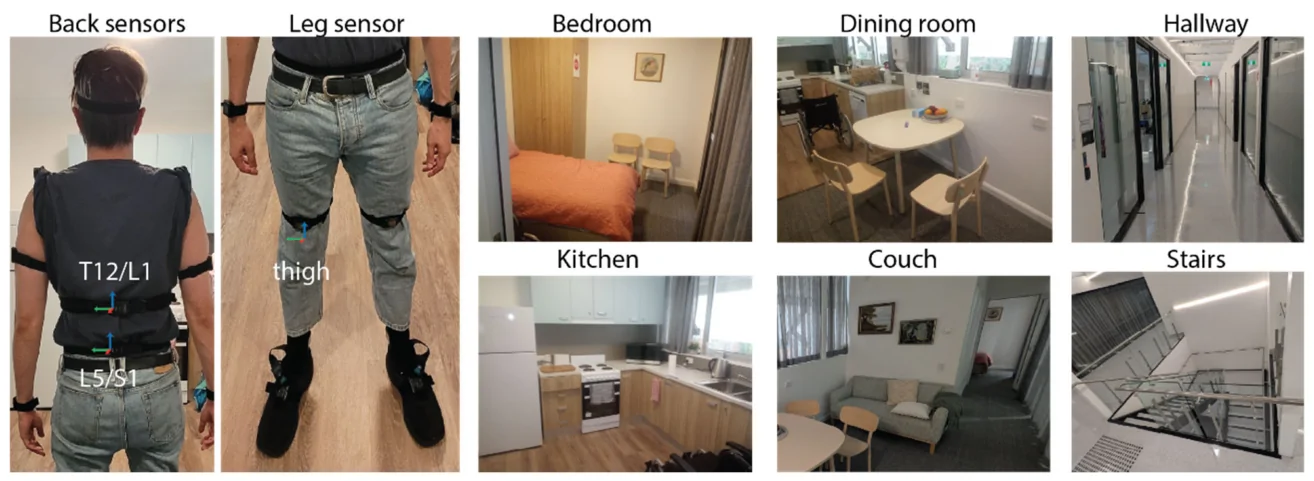
Pictures showing the placement of sensors on the participants and within the simulated-living apartment.

Participants performed a single circuit of 72 functional tasks while wearing IMUs (APDM, Opal IMUs, Portland, OR, USA) strapped to their bodies, recording at 100 Hz. Several sensors were used but only two on the back and one on the right thigh were used in the current work (Figure 1). Data collection occurred at the Simulation House at the St Lucia Campus of The University of Queensland. Ethics approval by the University of Queensland Ethics Committee and informed consent was obtained from all eight participants prior to the experiment. Data from five of the eight participants were analyzed. This facility is set up to replicate an apartment with a kitchen, dining table, couch, bathroom, and bedroom (Figure 1).

Video recordings (20 Hz) were used to verify the accuracy of time and category labels assigned to each movement. Prior to each trial, the experimenter provided instructions to the participant and set up a camera to record the participant during the task. At 3 s prior to prompting the participant to begin the task, the experimenter simultaneously initiated recording on the video camera and flagged the timepoint in the IMU data file (via a remote trigger).

The 72 tasks were designed to encompass a wide range of movements and postures that could be expected in daily living. They were sub-grouped into categories of upright, sitting, stepping, bending, state transitions, and lying. See Supplementary Materials S1 for a full list of each task together with the instructions provided prior to each task.

### 2.2. Data Processing

Data from the IMUs were downsampled to 20 Hz to be compatible with the video recordings. The data from each IMU were used to estimate the angle (relative to the world) of the body segment to which they were attached using a gradient descent filter optimized for a sampling frequency of 20 Hz [17].

To generate a skeletal model for task labelling, ball-in-socket joints were modelled between the right thigh and pelvis (hip), as well as between the pelvis and trunk (lower-lumbar joint). These kinematic constraints were imposed directly on the skeletal model. As mentioned earlier, it was assumed that there was no overall translation of the model’s centre-of-mass. Imposing zero-translation effectively keeps the model centred in the animation frame, and activities such as walking on straight ground appear like the participant walking on a treadmill. Although we experimented with translations, the drift in positions was significant and generally made it more difficult to interpret the animations. The reconstructed kinematics were not validated against a criterion measurement of body motion (for example, optical motion capture). The present study therefore addresses whether a reviewer can identify the activity being performed from the animation, not how accurately the animation reproduces the underlying joint motion.

### 2.3. Graphical User Interface for Labelling Tasks

**Figure 2:**
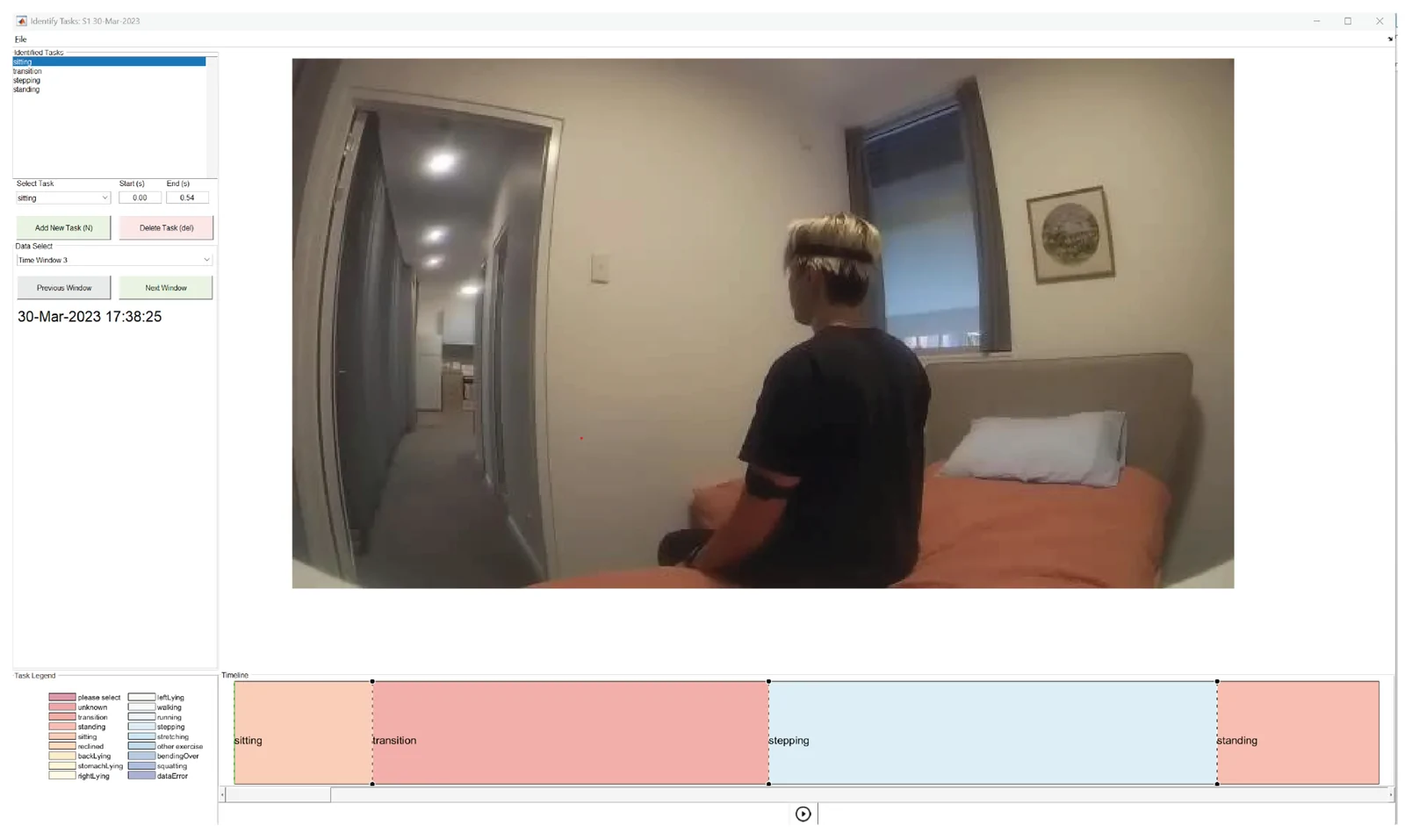
The graphical user interface used to label the tasks performed by the participant. In this panel, the video recording was shown, but an identical interface was used to label the IMU data.

**Figure 3:**
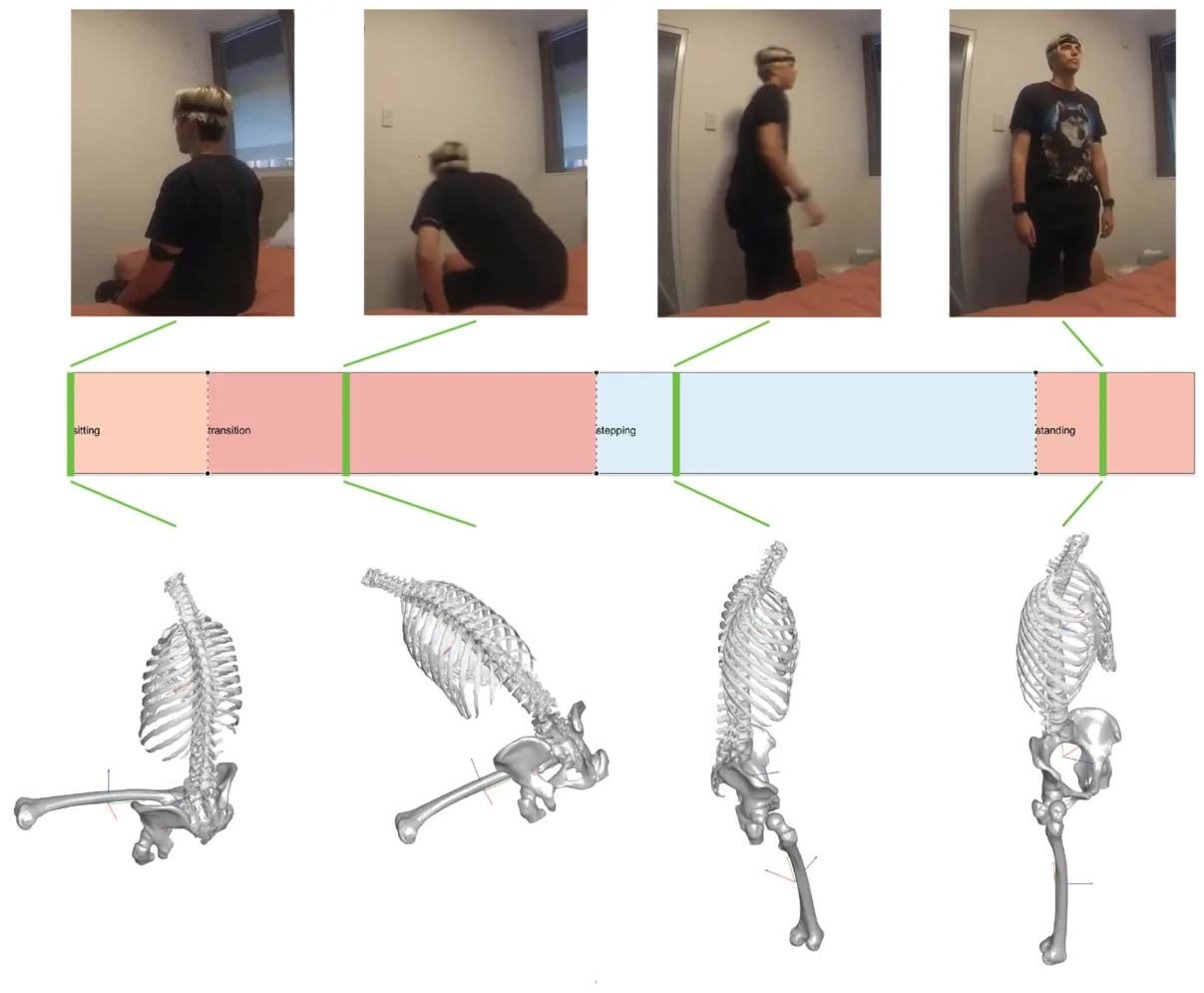
Keyframes of the video are shown for a representative trial along with the labels determined by the reviewer. The equivalent skeletal animation reconstructed from the IMU data is also shown in the bottom row.

A graphical user interface (GUI) was developed in Matlab 2023b (Mathworks, Natick, MA, USA) to aid the creation of labelled IMU data. The same GUI was used to label both the animated IMU data and the video recordings. A still picture of the GUI is found in Figure 2, and a video example of the GUI is found in Supplementary Materials S2. An example of the video and IMU animation for a representative trial is shown in Figure 3. Windows of time of about 10 s were then displayed to a reviewer, who was asked to label the window of time from a possible set of labels (Supplementary Materials S2). The reviewer was instructed to be as detailed as possible; there was no strict rule about how short a task could be, and multiple tasks could be labelled within each window.

If the reviewer was not strongly confident in what the skeletal animation represented, they were instructed to label those samples as “Unknown” to avoid incorporating incorrect labels into the dataset. Confidence was not quantified or recorded; it was a subjective judgement made by each reviewer. The reviewers were post-graduate scientists in the field of biomechanics. Each completed a one-hour training session in which the authors explained the labelling process and the software, after which the reviewers practiced labelling data while asking questions to clarify the process. They were also given a list of possible tasks to choose from when labelling the datasets (Supplementary Materials S1).

If multiple labels could apply to a given time point, the reviewers were instructed to choose the most specific label they were still strongly confident in. These hierarchies are detailed in Supplementary Materials S2 For stepping, reviewers were instructed to only label it as walking or running if it was a clear bout of either activity, or to simply label it as “stepping” if not (for example, taking a few shuffling steps in the kitchen tasks). Similarly, the label “bending over” could apply when the participant is sitting or standing and would supersede either of those labels in cases in which the reviewer determined that the label was appropriate.

For the labelling of transitions and shifts, they were instructed to only label those in which there was a clear start and ending of a motion, either within one state or as a transition between two states. If there was motion without a clear start and end point within a short window of less than 3 s, they were instructed to not label that as a transition.

### 2.4. Assessing Agreement of IMU-Based Labels

**Figure 4:**
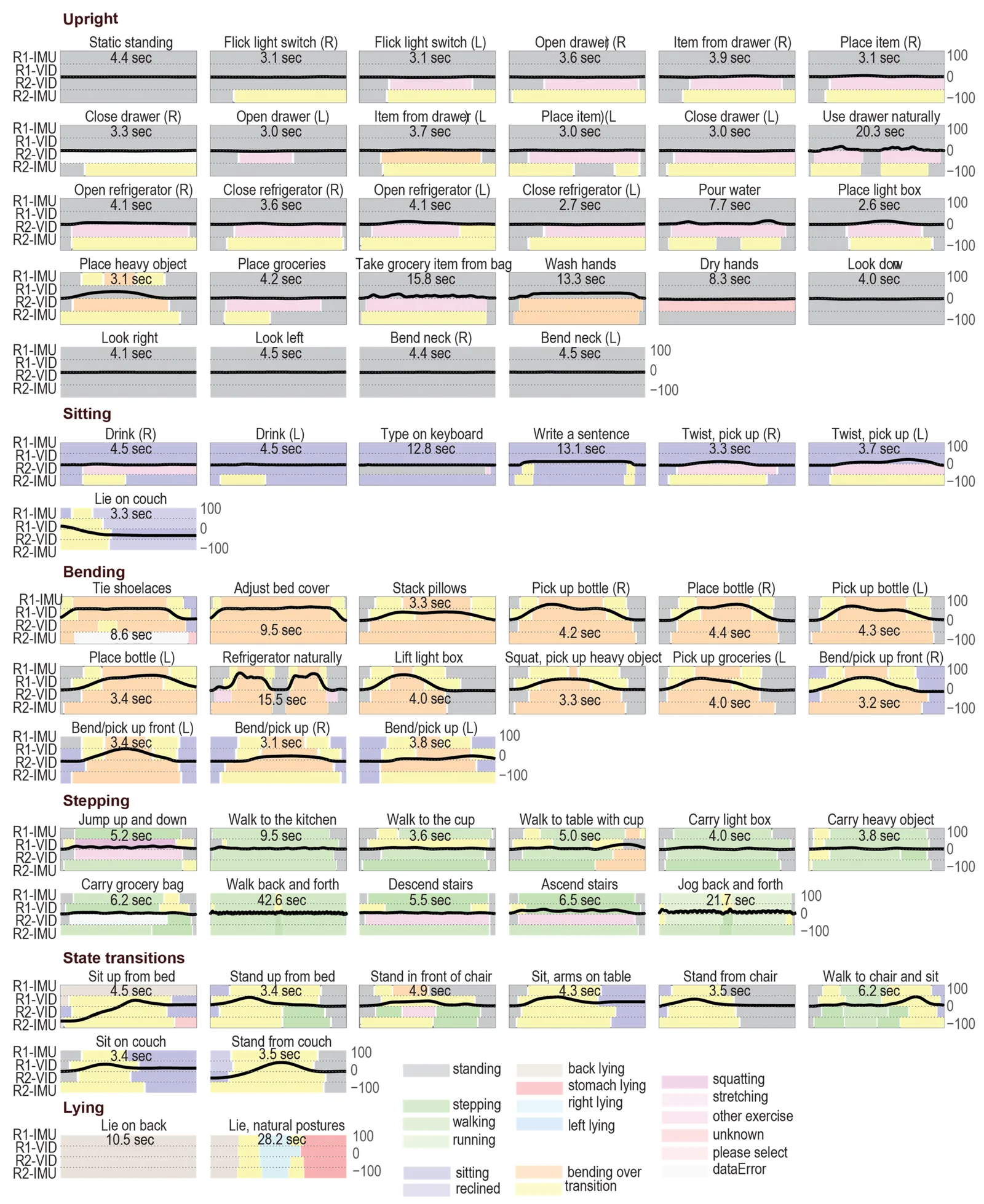
Labels generated by both reviewers (R1 and R2) for all tasks are shown for a representative participant (participant 3). The reviewers labelled the same data by observing both animations from IMU data and video. The background colour denotes the labels generated by the reviewers, broken into separate bars, each denoting a different set of labels. For example, “R1-IMU” denotes the labels generated by reviewer 1 by observing IMU data. The signal plotted (black solid line) is the inclination of the trunk segment. Intra-reviewer agreement can be visually assessed by comparing either the top two or bottom two bars, whereas inter-reviewer agreement can be observed by comparing the middle two or outer two bars.

Two reviewers were asked to label both the IMU and video data from the experiment. They were asked to review three sets of data, the first being a set of IMU animations from real-world living (not described in the present work), a second set of IMU animations from the simulated living experiment, and the third set of video recordings from the simulating living experiment. The reviewers were blinded to the fact that sets 2 and 3 were pulled from the same experiment. Additionally, the order of labelled windows was randomized (across each task, and for each reviewer separately) to minimize ordering effects. To further obfuscate the similarity between sets 2 and 3, a 0 to 10 s window (randomly decided) was added to both the beginning and end of the IMU animations from set 2. Figure 4 shows example data for a representative participant for all the tasks within each sub-grouping.

#### 2.4.1. Subsets of Labels

Agreement between labels was compared at three separate levels. Firstly, agreement for the full set of the most specific labels was compared. Secondly, these labels were collapsed to a more simplified set for comparison. Finally, both for the detailed and simplified labels, agreement was compared while including the “transition” label, versus not including the “transition” label, as a means of identifying what amount of error was simply due to disagreement over what constituted a transition between states and the start and end time of each transition, as opposed to errors only due to labelling a steady-state activity. This comparison is a sensitivity analysis: excluding transitions removes the most ambiguous samples, and simplifying the taxonomy reduces the number of classes, so both change the classification task and are expected to increase agreement. They are therefore not presented as improved performance on the same problem.

#### 2.4.2. Intra-Reviewer Agreement

To assess the feasibility of the proposed method, the labels for sets 2 and 3 (IMU vs. video) were compared for each reviewer against themselves. This intra-reviewer agreement was used to assess to what extent the IMU animations were able to convey enough information for the reviewer to accurately label the tasks. For each label, the video label was treated as the reference standard and agreement was quantified as the recall (true-positive rate, *TPR*) of the IMU-animation labels. Because recall does not account for false positives or chance agreement, we also calculated, for each label, precision and the F1-score (the harmonic mean of precision and recall). Summary metrics were the macro-averaged precision, recall (equivalent to balanced accuracy) and F1, in which every label contributes equally regardless of how often it occurred; the weighted-average F1, in which each label is weighted by its duration in the reference; and Cohen’s kappa (κ), a chance-corrected measure of agreement calculated over all samples. Macro-F1 was our primary summary metric because the label distribution was highly imbalanced. Because macro averages are sensitive to very rare labels, macro metrics are reported both over all labels and after excluding labels used for less than 30 s in total (pooled across participants) by either reviewer. For a given label L and reviewer R, the intra-reviewer recall ${TPR}_{intra}^{R,L}$ was defined as

(1) $${TPR}_{intra}^{R,L}=\frac{N_{IMU}^{R,L}}{N_{VIDEO}^{R,L}}\times 100\%$$

where $N_{VIDEO}^{R,L}$ is the total number of samples annotated by reviewer R for label L across all trials for a given participant, and $N_{IMU}^{R,L}$ is the number of those samples that were also labelled as the same label when the reviewer annotated the IMU animations. These metrics were calculated for each participant and reviewer and are reported as the mean ± standard deviation across the ten participant–reviewer pairs (five participants × two reviewers). They were calculated for both the full set of labels as well as the simplified set of labels. To assess more generally how likely a reviewer was to confuse one label with another when annotating IMU animations vs. video recordings, a confusion matrix was also calculated.

#### 2.4.3. Inter-Reviewer Agreement

To assess how the proposed methodology depends on the decision-making and judgement of individual reviewers, we also compared the labels across both reviewers for both the video-annotated labels and the IMU-animation labels. The primary outcome is a confusion matrix showing how likely (in the total number of labelled samples) the two reviewers were to label data as the same task, as compared to having disagreement about the task. Agreement was also quantified with the same metrics as in Section 2.4.2 (macro-F1, weighted F1 and κ; F1 and κ are symmetric between the two reviewers), calculated for each participant and summarized as the mean ± standard deviation across the five participants. To compare like with like, both modalities were restricted to the time samples covered by both the video and the IMU animation, which excludes the random padding added to the beginning and end of the animations.

## 3. Results

### 3.1. Intra-Reviewer Agreement: Skeletal Animation vs. Video Reference Standard

To evaluate the proposed annotation method, labels assigned by each reviewer using IMU-derived skeletal animations were compared with labels assigned by the same reviewer from synchronized video recordings, which served as the reference standard.

For the detailed label set, the macro-averaged F1-score across all labels was 61.9 ± 13.5% (68.3 ± 14.8% after excluding the rare labels reclined and squatting), the duration-weighted F1-score was 72.9 ± 10.0%, and Cohen’s κ was 0.67 ± 0.12 (Table 1). In a sensitivity analysis that removed all samples labelled as a transition by either reviewer, macro-F1 was 69.4 ± 10.9% (77.1 ± 12.6% excluding rare labels), weighted F1 was 83.1 ± 7.5% and κ was 0.78 ± 0.09. Because this analysis removes the most ambiguous samples, and therefore changes the task from labelling all movement to labelling steady-state activity, these values should not be interpreted as improved performance on the same problem. Rather, they indicate that much of the disagreement arose during brief changes between activities rather than during stable postures or movements.

Performance varied by activity class (Table 1). Back lying had the highest F1-score (90.2%), followed by standing (81.3%), sitting (80.6%), stomach lying (78.5%), right lying (76.7%), walking (75.1%), left lying (74.6%) and bending over (68.3%). Lower F1-scores were observed for the transition label (45.4%), stepping (43.9%), running (36.2%) and reclined postures (23.9%). Squatting showed no agreement (F1 = 0%), which likely reflects its very limited duration (0.5 s on average per participant–reviewer pair) and overlap with adjacent movement states.

### 3.2. Effect of Simplified Labels

To examine how label granularity affects agreement, labels were collapsed into four simplified classes: sitting, stepping, lying, and standing. Because this changes the classification task rather than measuring performance on the original one, results for the simplified set are not directly comparable to those for the detailed set.

For the simplified label set (with transition retained as a fifth label), macro-F1 was 78.0 ± 11.5%, weighted F1 was 80.4 ± 10.4% and κ was 0.74 ± 0.13. In the sensitivity analysis excluding samples labelled as transitions, macro-F1 was 92.8 ± 5.3%, weighted F1 was 92.0 ± 5.8% and κ was 0.88 ± 0.09. Among the activity classes, F1 was highest for sitting (87.5%) and lying (87.5%), followed by standing (83.7%) and stepping (81.8%) (Table 2); the transition label had the lowest F1 (49.6%).

These results indicate that agreement is higher when activities are classified at a broader behavioural level, although the simplified taxonomy is an easier classification problem than the detailed label set.

### 3.3. Misclassification Patterns

**Figure 5:**
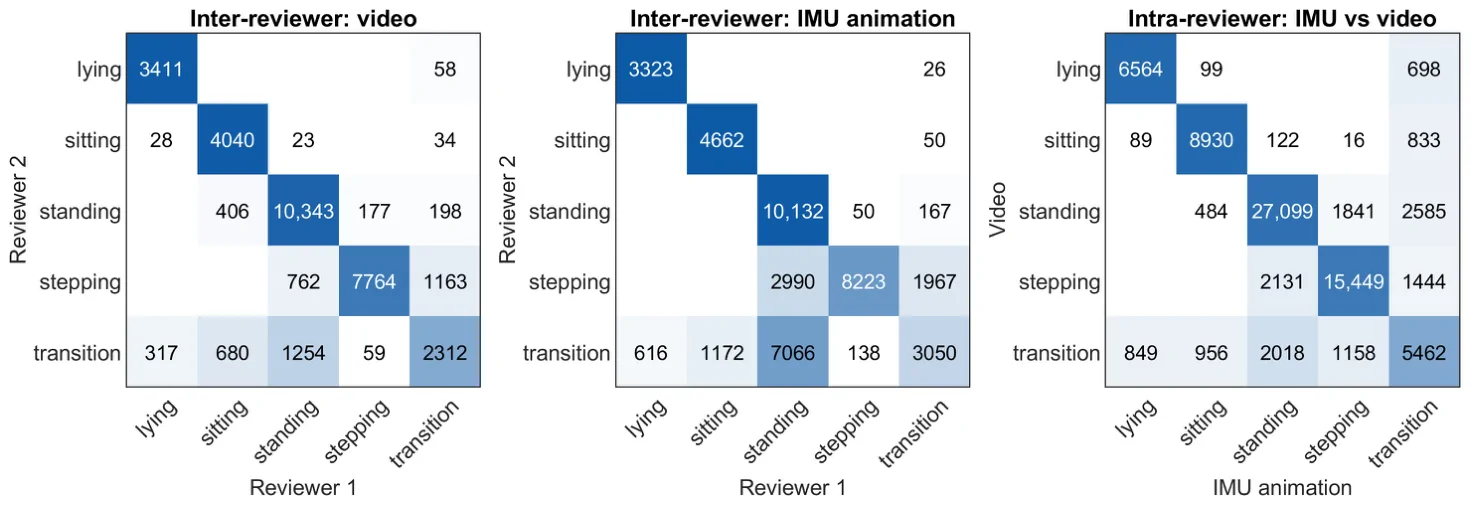
How often the simplified labels were confused with one another. Left: inter-reviewer confusion matrix for labels generated when the reviewers labelled the video. Middle: inter-reviewer confusion matrix for labels generated when the reviewers labelled the IMU animations. Right: intra-reviewer confusion matrix comparing labels generated from the IMU animations (columns) with labels generated from video (rows), summed across both reviewers. Numbers are counts of samples (20 Hz); shading shows the proportion of each row.

Confusion matrices demonstrated similar patterns for both intra-reviewer and inter-reviewer comparisons. The dominant source of disagreement was the classification of transition states. In many instances, one reviewer classified a brief interval as a transition, whereas the same period was classified by the other reviewer as sitting, standing, or stepping. Figure 5 shows the patterns of how the simplified tasks were misclassified across reviewers.

**Figure 6:**
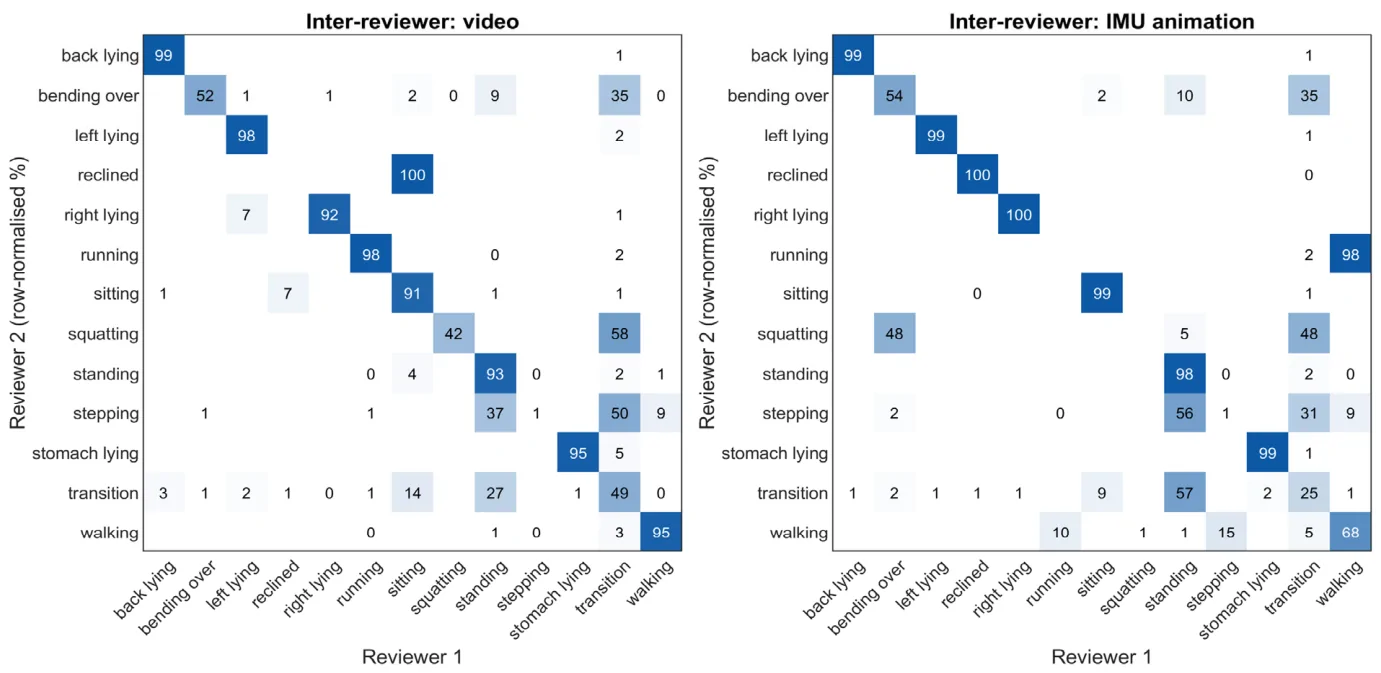
Confusion matrices for the detailed labels comparing the two reviewers across all participants (inter-reviewer agreement), for labels generated from video recordings (left) and IMU animations (right). Rows are reviewer 2 and columns are reviewer 1; each row is normalised to 100%, and blank cells contain no samples.

For the detailed label set, transitions were also the principal source of disagreement between reviewers when annotating the reference video recordings, suggesting that these disagreements were not unique to the skeletal animation method but instead reflect inherent ambiguity in defining precise movement boundaries. Figure 6 shows the confusion matrix of misclassification patterns across both reviewers for the detailed label set. This figure also shows how some labels (such as squatting, reclined, and stomach lying) only represent a very small portion of the collected data. Class-specific metrics for these infrequent labels are based on very few observations and are insufficient to support reliable conclusions regarding classification performance; they are reported for completeness only. The most frequent disagreement between reviewers was labelling a period as a “transition” as opposed to “bending over.”

### 3.4. Inter-Reviewer Agreement: Video vs. IMU Animations

Because the intended use of the method is for reviewers to label IMU animations without video, we also quantified agreement between the two reviewers when labelling the animations and compared it with their agreement when labelling video (Table 3; Figure 5 and Figure 6). For the detailed label set, inter-reviewer κ was 0.51 ± 0.06 for the animations and 0.72 ± 0.02 for video (macro-F1 61.3 ± 5.3% and 66.6 ± 4.1%, respectively). For the simplified label set, κ was 0.58 ± 0.06 for the animations and 0.80 ± 0.05 for video. Agreement between reviewers was therefore lower when labelling animations than when labelling video. Part of this difference was due to the labelling of transitions; when samples labelled as transitions were excluded the gap narrowed but remained, with κ of 0.74 ± 0.11 for the animations and 0.88 ± 0.05 for video (detailed set), and 0.86 ± 0.16 and 0.92 ± 0.03 (simplified set). For the animations, samples that reviewer 2 labelled as stepping or transition were frequently labelled as standing by reviewer 1, and samples that reviewer 2 labelled as running were labelled as walking by reviewer 1 (Figure 5 and Figure 6). With only two reviewers and five participants, these estimates should be interpreted cautiously.

## 4. Discussion

This study evaluated a novel human-in-the-loop method for generating reference labels from wearable IMU data using reconstructed skeletal animations. The principal finding was that reviewers were able to classify daily-life postures and movements from IMU-derived animations with substantial agreement (κ = 0.67 for the detailed label set) relative to synchronized video, which served as the reference standard. Intra-reviewer macro-F1 was 61.9% for the detailed label set and 78.0% for the simplified label set (κ = 0.74). Agreement was higher when transition samples were excluded (macro-F1 of 69.4% and 92.8%, respectively); however, this sensitivity analysis removes the most ambiguous samples and narrows the task, so it should not be read as improved performance on the same problem. Inter-reviewer agreement was lower when reviewers labelled animations (κ = 0.51 for the detailed label set) than when they labelled video (κ = 0.72). Together, these findings support the feasibility of using skeletal animation review as a privacy-preserving alternative to video-based annotation for developing activity-recognition datasets in a simulated living environment; their suitability for free-living IMU recordings remains to be tested.

### 4.1. Potential to Address a Major Barrier in Free-Living Biomechanics Research

The motivation for this work was the persistent lack of valid reference-labelled data for training machine-learning models using IMUs in real-world environments. Although IMUs are increasingly used to study movement outside the laboratory, most automated classifiers continue to rely on training datasets collected under controlled experimental conditions. Laboratory datasets offer precise labels because tasks are prescribed or directly observed, but they often fail to capture the complexity, variability, and spontaneity of real-world behaviour. Movements in daily life are shaped by context, furniture, terrain, fatigue, multitasking, and social demands, which are difficult to reproduce authentically in laboratory protocols. As a result, models trained exclusively on laboratory data may perform poorly when deployed in unconstrained settings.

The present findings suggest that annotation of IMU-derived skeletal motion may help bridge this gap. Rather than requiring cameras or laboratory supervision, this approach enables human review directly from the sensing modality used in field deployment. This may reduce the mismatch between training data and application data, while potentially preserving the ecological validity of real-world recordings (not tested here). In this regard, the method may be more than an annotation tool, namely a framework for generating realistic, labelled datasets for wearable activity recognition, pending validation in free-living recordings.

The usefulness of the methodology depends on how well the IMU animations recreate the general posture and motion of the body, and to what extent the lack of environmental context clues can affect agreement of labelling. This study could not separate these two factors, because the reconstructed kinematics were not independently validated. Based on the results for the relatively basic types of labels that were studied, it appears that even without any environmental cues, reviewers could still identify the general type of physical behaviour of the participants with substantial agreement relative to video, in this simulated, prescribed-task setting. It is important to separate two questions: whether the animation accurately reproduces the underlying movement, and whether a reviewer can correctly identify an activity from the animation. The present study addressed only the second.

### 4.2. Agreement for Common Daily-Life Behaviours

Agreement was highest for static and well-defined postures, particularly back lying, standing and sitting, with moderate agreement for walking and bending. These activities are characterized by distinctive segment orientations and movement patterns that are readily visible in skeletal reconstructions. For example, lying postures involve clear changes in trunk and thigh orientation relative to gravity, whereas walking generates rhythmic lower-limb motion that is visually identifiable. The agreement for these common behaviours is important because they represent many of the activity classes most relevant to health monitoring, ergonomics, rehabilitation, and musculoskeletal research.

In contrast, lower agreement was observed for running, stepping, reclined postures, and squatting. Several factors likely contributed. Some classes occurred infrequently, limiting the amount of available data and increasing the influence of single disagreements. Other tasks may share overlapping kinematic features with adjacent classes. For example, differentiating between squatting and sitting is difficult, while reclined postures may overlap with sitting or lying depending on trunk angle. Squatting was rare and we did not collect sufficient data to make strong conclusions about the feasibility of labelling it. These findings reinforce that classifier performance is influenced not only by signal quality, but also by the behavioural distinctiveness and prevalence of the target class.

### 4.3. Transition States Are Inherently Difficult to Label

The most consistent source of disagreement was the classification of transition periods. This was evident in both intra-reviewer comparisons (animation vs. video) and inter-reviewer comparisons using either video or animations. This is an important observation because it suggests that disagreement was not primarily caused by limitations of the skeletal animation method, but by the intrinsic ambiguity of defining when one activity ends and another begins.

Transitions in natural movement are often gradual rather than discrete. For example, the change from standing to sitting may involve anticipatory trunk flexion, weight transfer, descent, seat contact, and postural settling. Different reviewers may reasonably choose different temporal boundaries for the onset or completion of the same task. This issue is well recognized in human annotation more broadly and highlights that reference labels are often probabilistic rather than absolute.

For future work, explicit operational definitions of transitions, minimum dwell times, or hierarchical labelling approaches may improve consistency. An alternative is to treat transitions as a separate uncertainty class or to model activity continuously rather than as discrete windows. Importantly, agreement was higher after excluding transition samples, indicating that a large share of the disagreement arose at transition boundaries. This is expected, because these are the most ambiguous samples, so this sensitivity analysis should be read as characterizing the labelling of steady-state activity rather than as improved performance of the method.

### 4.4. Label Granularity Matters

Agreement was higher for the simplified label taxonomy. When detailed classes were collapsed into sitting, standing, stepping, and lying, macro-F1 increased from 61.9% to 78.0% (κ from 0.67 to 0.74), reaching 92.8% when transition samples were excluded. Because collapsing classes changes the classification task, this is not an improvement on the same problem. This finding has practical significance for the development of machine-learning systems.

Many real-world applications do not require highly granular labels. For example, surveillance of sedentary behaviour may only require discrimination between sitting, standing, and stepping. Similarly, monitoring recovery after surgery may focus on time spent walking, lying, or being upright. In these contexts, broader labels may provide more robust and clinically meaningful outputs than fine-grained taxonomies with lower reliability.

Conversely, some research questions may require detailed distinctions such as bending, lifting, or asymmetrical loading. The present findings suggest that such specificity is achievable for some tasks but may require clearer definitions, additional sensors, longer training of annotators, or probabilistic models. Thus, the label structure should be aligned with the intended scientific or clinical use case.

### 4.5. Advantages over Existing Reference-Labelling Approaches

This method addresses several limitations of current annotation paradigms. First, it avoids continuous video capture, reducing privacy concerns in homes, workplaces, and sensitive environments. Second, it preserves human oversight, allowing reviewers to identify unusual or novel behaviours that automated systems may miss. Third, because labels are generated from IMU-derived kinematics, the method may be more representative of deployment data than labels transferred from camera-based systems.

These advantages may be particularly relevant in biomechanics and rehabilitation research, where clinically meaningful behaviours often occur unpredictably over days or weeks. Examples include prolonged sitting posture, repeated bending exposure, guarded movement during pain flare-ups, altered sleep movement, or gradual changes in mobility during recovery. Such phenomena are difficult to capture comprehensively in laboratory settings.

### 4.6. Limitations

Several limitations should be acknowledged. First, the study used a simulated living environment rather than unrestricted free-living recordings. Although this improved realism relative to laboratory task trials, behaviour may still differ from fully natural settings. The results should therefore not be extrapolated to unrestricted free-living recordings, which involve unprompted behaviours, longer recordings, other populations and greater positional drift (translation was removed here), and which require separate validation. Second, only two reviewers, each with one hour of training, were included; larger studies are needed to characterize variability across annotators with different training backgrounds. Inter-reviewer variability, particularly when labelling the animations (Section 3.4), may limit the generalizability and reproducibility of the reported findings, because different reviewers, or different training procedures, may yield different labels. Third, the sensor set and skeletal model were intentionally simple. Additional sensors or richer biomechanical reconstructions may improve discrimination of complex tasks. The reconstruction was not validated against a criterion measure of segment motion, and translation was removed because of drift; agreement with video therefore reflects how well reviewers can identify activities from the animation rather than the kinematic accuracy of the animation. Fourth, some classes had low prevalence, limiting confidence in class-specific estimates. For classes represented by very few observations (e.g., squatting, reclined, and stomach lying), the corresponding class-specific metrics are insufficient to support reliable conclusions regarding classification performance. Fifth, video was treated as the reference standard, yet even video labels showed disagreement for transitions, underscoring that reference labels themselves are imperfect. Sixth, annotation time and reviewer workload were not recorded, so we could not evaluate whether IMU-based annotation is faster or less burdensome than video annotation. Whether the method scales to long recordings therefore remains to be tested in future work. Seventh, data from only five of the eight participants were analyzed, which was judged sufficient for this initial evaluation of agreement; future work could explore how agreement varies with differences in how people execute tasks in a larger testing setting.

Finally, there is only a limited amount of information about the environment and how the avatar moves through space. The sensors are unable to detect the nature of the person’s environment and so this information is not available to the reviewer during labelling. This includes limitations in displaying how the avatar translates through space, since IMUs inherently struggle with positional drift over time. For this reason, visually determining how quickly someone is moving through space or if they are changing elevation is difficult. There are ways of improving this issue by including additional sensors or applying additional analytical assumptions (such as the zero-velocity foot correction during walking [18]) that can be explored.

### 4.7. Future Directions

Future research should evaluate this framework during multi-day free-living monitoring in homes, workplaces, and clinical populations. Semi-automated workflows in which algorithms propose labels for reviewer confirmation may further reduce annotation burden. Hierarchical labelling systems could distinguish posture, locomotion, task context, and uncertainty simultaneously. Because the assignment of “Unknown” labels relied on subjective reviewer confidence, future studies should investigate structured decision rules (for example, explicit criteria for when a label is considered sufficiently certain), reviewer calibration procedures (for example, a shared practice dataset with feedback and an agreement threshold before independent labelling), or other approaches aimed at reducing subjective variability between reviewers. Integration with self-reported notes and other data sources such as GPS tracking may also enable richer behavioural phenotyping. Additional work on improving sensor fusion algorithms to account for positional drift and magnetic interference could also improve accuracy.

### 4.8. Conclusions

IMU-derived skeletal animation review provides a practical method for generating reference labels from wearable sensor recordings. Agreement with video was substantial for common postures and locomotor activities, and it was highest for the simplified label set and when transition samples were excluded (sensitivity analysis). By combining privacy preservation and human interpretability, this approach may offer a pathway to develop training datasets for activity-recognition models in real-world biomechanics research, once validated in free-living recordings. The present evidence comes from a small, simulated, prescribed-task dataset with two reviewers; validation in free-living recordings remains to be done.

## Figures and Tables

**Table 1:** Agreement between the labels reviewers generated from IMU animations and their own labels from video (reference standard), detailed label set. Values are mean ± SD across ten participant–reviewer pairs; ^†^ labels used for less than 30 s in total (excluded from the second macro-average). The final column repeats F1 after excluding samples labelled as transitions by either reviewer (sensitivity analysis). Entries marked – denote calculations that are not applicable.

Label	Reference Duration (s)	Recall (%)	Precision (%)	F1 (%)	F1, Transitions Excluded (%)
back lying	15.3 ± 4.5	95.6 ± 6.3	87.8 ± 19.0	90.2 ± 11.1	96.7 ± 4.9
bending over	54.5 ± 16.8	66.8 ± 29.5	71.7 ± 21.8	68.3 ± 26.5	75.6 ± 27.3
left lying	8.0 ± 6.9	73.6 ± 33.2	76.3 ± 35.9	74.6 ± 34.1	83.5 ± 33.5
right lying	6.0 ± 2.2	74.6 ± 31.4	81.1 ± 32.5	76.7 ± 30.5	90.0 ± 31.6
running	17.2 ± 5.1	34.5 ± 45.0	38.2 ± 49.5	36.2 ± 47.1	38.3 ± 49.5
sitting	50.7 ± 14.5	79.1 ± 10.5	84.3 ± 14.1	80.6 ± 8.2	87.1 ± 7.2
squatting ^†^	0.5 ± 0.9	0.0 ± 0.0	0.0 ± 0.0	0.0 ± 0.0	0.0 ± 0.0
standing	163.9 ± 63.6	78.6 ± 16.6	87.9 ± 12.5	81.3 ± 10.3	88.0 ± 7.9
stepping	16.3 ± 7.1	46.1 ± 26.7	49.2 ± 27.3	43.9 ± 22.2	47.4 ± 22.7
stomach lying	7.4 ± 6.3	77.5 ± 32.6	79.8 ± 33.8	78.5 ± 32.9	86.9 ± 35.1
transition	55.8 ± 22.4	56.8 ± 35.2	40.0 ± 16.6	45.4 ± 23.8	–
walking	62.3 ± 6.8	74.0 ± 14.1	78.3 ± 11.3	75.1 ± 10.2	77.9 ± 10.6
reclined ^†^	1.7 ± 2.6	50.8 ± 38.4	23.5 ± 36.2	23.9 ± 36.2	29.0 ± 42.2
**Macro-average, all labels**	**–**	**67.0 ± 15.8**	**63.8 ± 12.7**	**61.9 ± 13.5**	**69.4 ± 10.9**
**Macro-average, rare labels ^†^ excluded**	**–**	**69.0 ± 14.5**	**70.5 ± 14.1**	**68.3 ± 14.8**	**77.1 ± 12.6**
**Weighted average (by duration)**	**–**	**73.4 ± 8.6**	**75.2 ± 11.3**	**72.9 ± 10.0**	**83.1 ± 7.5**
**Cohen’s κ**	**–**	**–**	**–**	**0.67 ± 0.12**	**0.78 ± 0.09**

**Table 2:** Agreement between the labels reviewers generated from IMU animations and their own labels from video (reference standard), simplified label set. Values are mean ± SD across ten participant–reviewer pairs. The final column repeats F1 after excluding samples labelled as transitions by either reviewer (sensitivity analysis). Entries marked – denote calculations that are not applicable.

Label	Reference Duration (s)	Recall (%)	Precision (%)	F1 (%)	F1, Transitions Excluded (%)
sitting	49.9 ± 15.2	88.9 ± 3.9	86.6 ± 12.5	87.5 ± 8.1	95.3 ± 6.4
stepping	95.1 ± 10.9	80.8 ± 17.9	86.2 ± 12.9	81.8 ± 12.8	87.3 ± 12.3
lying	36.8 ± 10.2	87.8 ± 12.4	87.7 ± 17.5	87.5 ± 14.7	97.8 ± 4.9
standing	160.0 ± 64.1	80.8 ± 16.1	89.9 ± 10.6	83.7 ± 9.7	90.8 ± 7.0
transition	52.2 ± 21.7	60.9 ± 37.8	44.5 ± 17.3	49.6 ± 25.8	–
**Macro-average, all labels**	**–**	**79.8 ± 11.9**	**79.0 ± 10.8**	**78.0 ± 11.5**	**92.8 ± 5.3**
**Weighted average (by duration)**	**–**	**80.9 ± 9.1**	**82.5 ± 10.8**	**80.4 ± 10.4**	**92.0 ± 5.8**
**Cohen’s κ**	**–**	**–**	**–**	**0.74 ± 0.13**	**0.88 ± 0.09**

**Table 3:** Inter-reviewer agreement for labels generated from video and from IMU animations, restricted to the same time samples. Values are mean ± SD across five participants; ^†^ labels used for less than 30 s in total by either reviewer (excluded from the second macro-average).

Modality, Label Set	Macro-F1 (%)	Macro-F1, Rare Labels ^†^ Excluded (%)	Weighted F1 (%)	κ	κ, Transitions Excluded
Video, detailed	66.6 ± 4.1	81.7 ± 2.1	75.4 ± 2.1	0.72 ± 0.02	0.88 ± 0.05
IMU animation, detailed	61.3 ± 5.3	58.8 ± 3.5	54.5 ± 5.9	0.51 ± 0.06	0.74 ± 0.11
Video, simplified	83.2 ± 3.3	83.2 ± 3.3	84.3 ± 4.7	0.80 ± 0.05	0.92 ± 0.03
IMU animation, simplified	71.5 ± 4.8	71.5 ± 4.8	64.9 ± 5.1	0.58 ± 0.06	0.86 ± 0.16

## Data Availability

The original data presented in the study are openly available in FigShare, DOI: https://doi.org/10.6084/m9.figshare.32873096.

## Supplementary Materials

The following supporting information can be downloaded at: https://www.mdpi.com/article/10.3390/s26216683/s1, Supplementary Materials S1: task list and instructions for simulated living experiment; Supplementary Materials S2: Task definitions and hierarchy.

## Abbreviations

The following abbreviations are used in this manuscript:

IMU	Inertial Measurement Unit
GUI	Graphical User Interface
