# Supplementary material for: A Human-in-the-Loop Reference-Label Curation Framework for Task Classification During Free-Living, Using 3D Animation of IMU Data
Source: Sensors (Basel). 2026 Oct 22;26(21):6683. doi: 10.3390/s26216683 (PMC13766114; doi:10.3390/s26216683)
Supplement: Supplementary file 1 [file sensors-26-06683-s001.zip › Supplementary Materials S2 - task definition.pdf]

Supplementary Materials S2 – Task definitions and hierarchy

The reviewers were asked to label each period of time displayed to them, being asked to prioritize labels that are more specific if possible. The simplified labels were collapsed *post hoc* for analysis based on the relationship between the label in the first column of the table, and the simplified label in the second column of the table. Any specific instructions are listed in the third column.

Label	Simplified label	Additional instruction
**Standing**	Standing	
**Sitting**	Sitting	Any sitting that is not clearly reclined
**Reclined**	Sitting	
**Back lying**	Lying	
**Stomach Lying**	Lying	
**Right Lying**	Lying	
**Left Lying**	Lying	
**Stepping**	Stepping	Any stepping that is not clearly walking or running
**Walking**	Stepping	
**Running**	Stepping	
**Transition**	Transition	Any non-instantaneous period in which it is clear there is a transition from one label to another
**Bending over**	Other	
**Squatting**	Other	
**Stretching**	Other	
**Other exercise**	Other	Any movement sequence in which it is clear the person is engaging in some kind of other exercise not encompassed by the other labels
**Unknown**	Other	Any period in which the reviewer is not strongly confident they can assign one of the other labels
**Please select**	Other	The default label, demarks if the reviewer failed to complete the task for that instance
**Data error**	Other	If there is clearly some kind of interference and the animation is uninterpretable
