# Supplementary material for: A Human-in-the-Loop Reference-Label Curation Framework for Task Classification During Free-Living, Using 3D Animation of IMU Data
Source: Sensors (Basel). 2026 Oct 22;26(21):6683. doi: 10.3390/s26216683 (PMC13766114; doi:10.3390/s26216683)
Supplement: Supplementary file 1 [file sensors-26-06683-s001.zip › Supplementary Materials S1 - task list.pdf]

Supplementary Materials S1 – task list and instructions for simulated living experiment

The participants were asked to complete a series of tasks in an apartment with the instructions provided below.

	Task Name	Instructions
**0**	Static standing	Stand upright with arms relaxed by your sides.
**1**	Jump	Using both legs, do five jumps, attempting to land back in the same spot you started.
**2**	Sit up from bed	Starting from lying on your back, sit up and sit with your legs over the edge of the bed. When sitting, keep your hands at your sides or place them on your thighs.
**3**	Tie shoelaces	Bend forward to tie up a shoelace. Keep your feet on the floor. When you are done sit straight up, and place your hands at your sides or on your thighs.
**4**	Stand up, face bed	Stand up from the bed and turn around 180 degrees to face the bed. Keep your arms at your sides.
**5**	Adjust bed cover	Cover the bed with the sheet standing in one place somewhere in the middle of the bed. Please use both hands and try and stand on the same spot.
**6**	Stack the pillows	Place the pillows in a neat stack at the head of the bed standing at one place somewhere in the middle of the bed. Please use both hands to hold each pillow.
**7**	Switch light right	Use your right hand to switch on/off the light switch on the wall.
**8**	Switch light left	Use your left hand to switch off/on the light switch on the wall.
**9**	Walk (<10m) to kitchen bench	Walk at your natural pace to the shelves in the kitchen.
**10**	Open shelf door (right arm)	Open the door of the high shelf with your right hand. Keep your left hand at your side or in your pocket. When done place your arm to your side.
**11**	Take an item from shelf (right arm)	Use your right hand to take one item from the shelf and place it on the bench. When done place your arm to your side.
**12**	Place item in shelf (right arm)	Use your right hand to take the item from the bench and return it to the shelf. When done place your arm to your side.
**13**	Close shelf door (right arm)	Close the door of the high shelf with your right hand. Keep your left hand to your side or in your pocket. When done place your arm to your side.
**14**	Open shelf door (left arm)	Open the door of the high shelf with your left hand. Keep your right hand at your side or in your pocket. When done place your arm to your side.
**15**	Take an item from shelf (left arm)	Use your left hand to take one item from the shelf and place it on the bench. When done place your arm to your side.
**16**	Place item in shelf (left arm)	Use your left hand to take the item from the bench and return it to the shelf. When done place your arm to your side.
**17**	Close shelf door (left arm)	Close the door of the high shelf with your left hand. When done place your arm to the side.

**18**	Shelf task naturally	Using either hand take five items, place them on the bench, move them back to the shelf and close the shelf
**19**	Open fridge (right hand)	Open the door of the fridge with your right hand. Keep your left hand at your side or in your pocket.
**20**	Pick bottle (right hand)	Bend at the hips and use your right hand to take a bottle from the bottom shelf and place it on the bench. Do not squat with your legs.
**21**	Place back bottle (right hand)	Take the item from the bench, bend down and place it back in the fridge.
**22**	Close fridge (right hand)	Close the fridge door with your right hand. Keep your left hand in your pocket or simply let it hang at your side.
**23**	Open fridge (left hand)	Open the door of the fridge with your left hand. Keep your right hand at your side or in your pocket.
**24**	Pick bottle (left hand)	Bend at the hips and use your left hand to take a bottle from the bottom shelf and place it on the bench. Do not squat with your legs.
**25**	Place back bottle (left hand)	Using your left hand take the item from the bench, bend down and place the items back in the fridge.
**26**	Close fridge (left hand)	Close the door of the fridge with your left hand. Keep your right hand at your side or in your pocket.
**27**	Fridge task naturally	Use either or both hands to open the fridge door, move two bottles to the bench, wait for a couple of seconds, and place the items back in the fridge, and close the fridge.
**28**	Walk to the cup	Walk to stand in front of the bench where the cup is.
**29**	Pour into cup	Hold the cup by its handle with your left hand and pour liquid from the jug into the cup with your right hand. When done, place the jug on the bench, and continue to hold the cup and the jug.
**30**	Walk to table with cup	While holding just the cup by its handle with the left hand, walk to the table and place the cup on the table, and rest your arms at your sides when done.
**31**	Pull chair and stand	Pull out the chair with your right hand and stand in front of the chair.
**32**	Sit down with arms on table	Sit naturally with your arms resting on the table.
**33**	Drink once (right arm)	Hold the cup with your right hand and pretend to drink once.
**34**	Drink once (left arm)	Hold the cup with your left hand and pretend to drink once.
**35**	Type a sentence	Type the following sentence on the keyboard "The quick brown fox jumps over the lazy dog."
**36**	Writing a sentence	Pick up the pen and write "Write with a pen" on the piece of paper. "The quick brown fox jumps over the lazy dog."
**37**	Stand from chair	Stand up from the chair.
**38**	Lift light box	Without squatting, bend at the hips and lift up the light box with both arms.
**39**	Carry light box	While carrying the box, walk over to the table.
**40**	Place light box on table	Place it on the table and let go.

**41**	Squat pick up heavy object	Squat and lift up the heavy object with both arms
**42**	Carry heavy object	While carrying the object, walk over to the table.
**43**	Place heavy object on table	Place it on the table and let go.
**44**	Pick up grocery bag (left hand)	Without squatting, bend at the hips and grab the grocery bag with your left hand, and carry it to the kitchen bench.
**45**	Carry grocery bag	Carry it to the kitchen bench and wait in front of the bench.
**46**	Place grocery bag on kitchen bench	Place the grocery bag up on the bench close to the edge, and let go.
**47**	Take item out of grocery bag	Use your left hand to keep the bag open and use your right hand to remove all items from the bag and place them on the bench to your right side.
**48**	Walk to chair and sit	Walk back to the chair and sit down at the chair.
**49**	Bend pickup (right front)	Bend over to pick up the apple on the floor in front of you with your right hand, and bring it to your thigh. Keep your left hand on your thigh.
**50**	Bend pickup (left front)	Bend over to pick up the orange on the floor in front of you with your left hand, and bring it to your thigh. Keep your right hand on your thigh.
**51**	Bend pickup (right side)	Bend to the right to pick up the apple on the floor to your right with your right hand, and bring it to your thigh. Keep your left hand on your thigh.
**52**	Bend pickup (left side)	Bend to the left to pick up the orange on the floor to your left with your left hand, and bring it to your thigh. Keep your right hand on your thigh.
**53**	Twist pickup (right)	By twisting your torso, pick up the object to your right with your left hand, and bring it to your thigh.
**54**	Twist pickup (left)	By twisting your torso, pick up the object to your left with your right hand, and bring it to your thigh.
**55**	Sit on couch	Sit on the couch.
**56**	Lie on couch	Lie back into a relaxed posture on the couch.
**57**	Stand up from the couch	Stand up from the couch.
**58**	Simulate wash hand 10 sec	Simulate turning on the tap with your left hand, placing your right hand on the bench, and then simulate washing your hands, and then turn off the tap with the left hand.
**59**	Simulate dry hand	Pretend you are wiping your hands dry.
**60**	Look down	While standing, bend your neck forward to look down at your feet.
**61**	Look to the right	While standing, turn your neck to look over your right shoulder.
**62**	Look to the left	While standing, turn your neck to look over your left shoulder.
**63**	Bend neck to the right	While standing, bend your neck to the side to get your right ear close to your right shoulder.
**64**	Bend neck to the left	While standing, bend your neck to the side to get your left ear close to your left shoulder.
**65**	Walk straight and back	Walk down to the end of the hallway.

**66**	Descend stairs	Walk down this flight of stairs.
**67**	Ascend stairs	Walk back up this flight of stairs.
**68**	Jog/Run and back	Jog along this hallway.
**69**	Jump	Using both legs, do five jumps, attempting to land back in the same spot you started.
**70**	Lying on bed on back.	Lie on the bed, on your back. Do not cross your legs, keep your hands at your sides or on your chest/stomach.
**71**	Sleep different natural postures	Start lying on your back, and then roll over to your side. Hold each position for 10 seconds.
